# Italian program for independent research on drugs: 10 year follow-up of funded studies in the area of rare diseases

**DOI:** 10.1186/s13023-016-0420-4

**Published:** 2016-04-12

**Authors:** Giuseppe Traversa, Lucia Masiero, Luciano Sagliocca, Francesco Trotta

**Affiliations:** Pharmacoepidemiology Unit, National Centre for Epidemiology, National Institute of Health, Viale Regina Elena 299, 00161 Rome, Italy; Italian National Transplant Center, National Institute of Health, Viale Regina Elena 299, 00161 Rome, Italy; ARSan Agenzia Regionale Sanitaria Regione Campania, Centro Direzionale di Napoli, Isola F9, 80143 Naples, Italy; Department of Epidemiology, Lazio Regional Health Service, Via Cristoforo Colombo 112, 00147 Rome, Italy

**Keywords:** Independent research, Rare diseases, Clinical trials, Cohort studies, Bibliometrics

## Abstract

**Background:**

In 2005 the Italian Medicines Agency (AIFA) started a program on independent research on drugs, with the aim to promote clinical research in areas of limited commercial interest. For 3 years (2005–2007) an area of the program was reserved to studies in the field of rare diseases. There is a concern that public funding of research may be wasted. We investigated the outcome of the program.

**Methods:**

We conducted a cohort study on the projects that were funded by the AIFA in the area of rare diseases. The outcomes were the proportion of published studies, time to publication, impact factor of the publishing journals and relevance for clinical practice. We retrieved published articles through a literature search in peer reviewed biomedical journals indexed by Pubmed. We used the Kaplan–Meier method to estimate the cumulative probability of publication by time from project starting to publication.

**Results:**

During the period 2005–2007, 62 projects were funded in the area of rare diseases. Most of the studies (n 39; 63 %) had a randomized design and in 22 (35 %) the control group received an active treatment. For 39 studies (63 %) we retrieved a publication in a peer reviewed journal. The median time to publication was 74 months and, at the maximum period of follow up (109 months), the cumulative probability of publication reached 77 %. The median impact factor was 5.4 (range 1.4–52.4). Considering the clinical relevance, more than 30 % of the published articles presented conclusive findings; an additional 10 % of the studies reached potential breakthrough findings.

**Conclusions:**

Even though it takes time to set up and conduct a funding program for independent research on drugs, the results are highly rewarding. Independent funding is crucial in supporting studies aimed at answering questions that are relevant for clinical practice despite the lack of sufficient commercial interest.

**Electronic supplementary material:**

The online version of this article (doi:10.1186/s13023-016-0420-4) contains supplementary material, which is available to authorized users.

## Background

In a world of limited resources, research investments need to compete with alternative purposes. To oppose the suggestion of wasting public funding, [1, 2] researchers are required to demonstrate that they are acting in the best interest of the society: focusing on relevant issues, collaborating and sharing information to exploit as far as reasonable the acquired data [3, 4], and publishing all the available findings [5]. Documenting how funding programs are managed and which results are obtained should also be considered a responsibility of the researchers’ community.

In 2005 the Italian Medicines Agency (AIFA) started a program on independent research on drugs, with the aim to promote clinical research in areas of limited commercial interest [6, 7]. Finance for this program comes through an innovative policy: all international and national pharmaceutical companies operating in Italy are required to contribute 5 % of their yearly expenditure for promotional initiatives to a national fund. For 3 years (2005–2007) an area of the program was reserved to studies in the field of rare diseases. The aim was to fund clinical studies to acquire additional information on the benefit-risk profile of orphan drugs (either approved by the European Medicines Agency, EMA, or holding an “orphan drug designation”), and of drugs that were used off-label in the treatment of rare diseases. Overall, 64 projects were approved in this area, for a total of 13.7 million euro [6, 7].

The call in the area of rare diseases was subsequently discontinued and the entire program, after three successive calls (in 2008, 2010 and 2012), is currently on hold [8]; the call launched in 2012 was finalized in March 2016. Various reasons may explain the suspension of the program as well as the exclusion of the rare diseases area. Among the others, some skepticism about the role and the quality of independent research was present.

We deemed of interest for the community of researchers and clinicians to document the results that can be achieved by funding independent research, also considering that relatively few examples are available. The objective of the present paper is to assess the outcome of the projects that were funded by AIFA in the field of rare diseases in terms of study completion, publications and potential implications for clinical practice.

## Methods

### Study design and setting

We conducted a retrospective cohort study based on all projects that were approved for funding between 2005 and 2007 in the area of rare diseases in the AIFA program for independent research on drugs. Details about the characteristics of the program have been presented elsewhere [6].

Each project was characterized in terms of: principal investigator; year of the call; year of the contract; study design (experimental vs observational); presence/absence of a control group; presence/absence of randomization; comparator for the primary outcome (placebo vs active treatment); number of patients included; clinical area. We adopted as starting point of the project the date in which the contract between AIFA and the institution of the principal investigator was signed. The list of project titles and principal investigators was published previously [6]; the starting date was publicly available through the AIFA website; further characteristics of the funded studies (e.g., study design and planned sample size) were obtained from two abstract books in which the initial organization of the studies was presented in two follow-up meetings held in 2008 and 2009 [9, 10].

### Ascertainment of the publications

We carried out a literature search in peer reviewed biomedical journals indexed by Pubmed using the name of the principal investigator together with keywords extracted from the project title. Two authors (FT, GT) carried out the selection of the publications matching project and publication titles. The full papers were retrieved and searched for an explicit reference to the funding of the research project by AIFA. In a few cases, no mention of the funding source was present. In these instances, a publication was accepted when the following three criteria were all met: the principal investigator of the research project was one of the authors; project and paper titles were matching; and the content of the paper was the same already presented in the abstract books. The publication status was ascertained after the starting point of the project up to 30 November 2015. In one case, we also accepted the personal communication by one investigator of a funded project indicating that a paper presenting the final results of the study had just been submitted for publication.

When no publication was retrieved, two additional exploring activities were carried out. First, the project title was searched in Google to verify if any publication was mentioned (other than those included in the abstract book previously cited). Second, we searched the archive of ClinicalTrials.Gov (CTG) and, when available, the website of the project to verify if any information was provided about the termination (prematurely or not) of the study, or the possibility that the study was, for different reasons, to be completed. We accepted as “ongoing” a study with an updated profile mentioning that the completion was expected not before the beginning of 2016.

### Relevance of the publications

For each article, the impact factor (IF) of the publishing journal was obtained consulting the Journal Citation Reports [11]. All IFs refer to the 5 year average 2009–2014.

The full text of each paper was analyzed to assess the potential relevance of the findings in terms of type of outcome (clinical vs surrogate) and implications for clinical practice. As for the implications, the published articles were categorized according to the conclusions stated by the authors as: 1) “potentially breakthrough”, when the study findings might radically modify the clinical prognosis of a disease or, in areas with previous uncertainties, the findings demonstrated that one of the compared options was drastically better than the other; 2) “conclusive-positive” (beneficial) results, when the study results presented in the article confirmed an option that was previously considered as part of the armamentarium for the treatment of a disease; 3) “conclusive-negative” (unfavorable) results, when the study results presented in the article disproved an option that was previously considered as part of the armamentarium for the treatment of a disease; 4) “potentially beneficial and proof of concept”, when the study results were presented in the article as encouraging and deserving further investigation.

### Statistical analysis

We described the main characteristics of the funded projects and of the published studies in terms of: prevalence of the condition; study design (presence of control group; presence of randomization); clinical area (oncology; congenital/genetic diseases; immune system disorders; others); age of the study population; planned and actual enrolment.

Categorical variables (e.g., type of control group) were reported as frequencies and percentages, and continuous variables (e.g., impact factor) as median and interquartile ranges (IQR). We used the Kaplan–Meier method to estimate the cumulative probability of publication by time from project starting to publication, with sub-analyses by elements of the study design (e.g., randomization, sample size, outcomes); the observation was censored on 30 November 2015. Log-rank test was performed to compare cumulative probability distributions. Logistic regression was used to estimate the association (Odds Ratio, OR) between study characteristics and the probability of publishing articles with greater relevance for clinical practice.

The statistical analysis was performed using STATA software (version 12.1; StataCorp LP, College Station, TX, USA). *P* < 0.05 (2-sided) was set as level of significance.

## Results

### Characteristics of funded and published studies

**Fig. 1:**
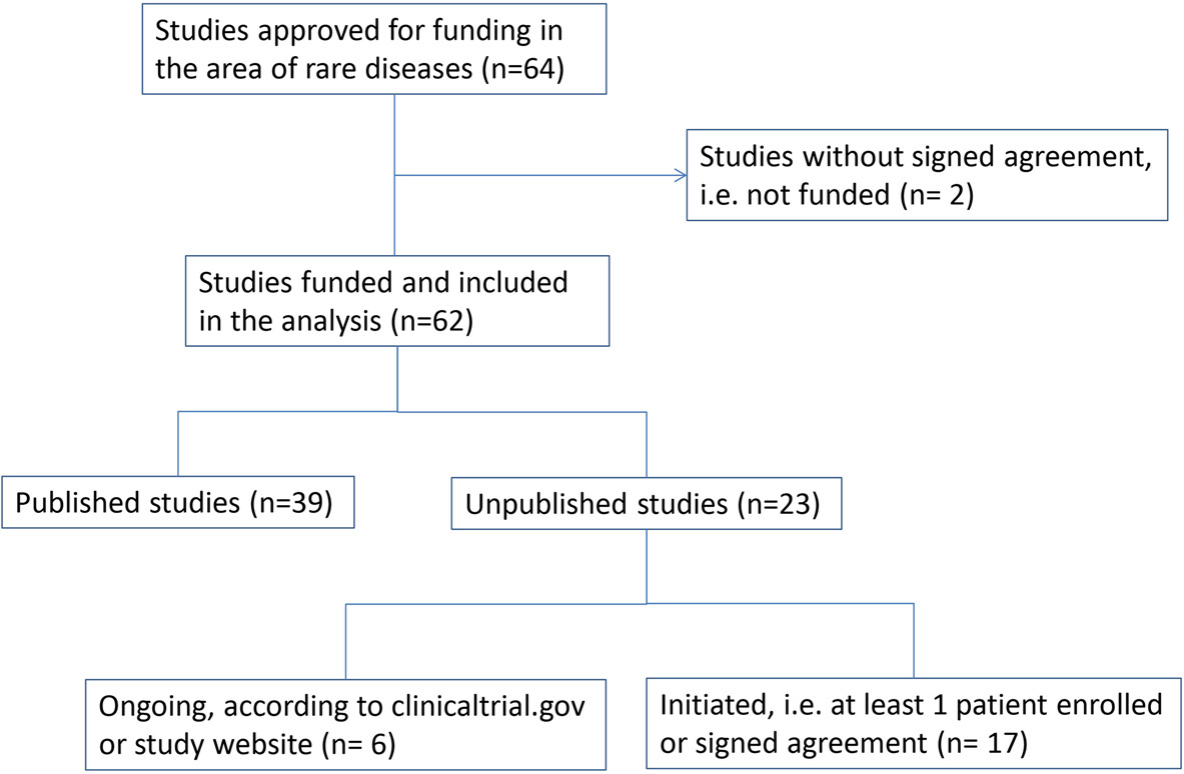
Flow chart of the studies approved for funding in the area of rare diseases included in the analysis

Two of the 64 projects approved for funding during the period 2005–2007 were not eligible for the analysis since the initial contract was never signed, leaving a sample of 62 studies (Fig. 1). For 63 % of the projects (39/62) at least one publication on a peer-reviewed journal was retrieved (Additional file 1: Table S1), whereas the remaining ones (23/62) were unpublished up to November 2015.

The main characteristics of the published and unpublished projects are reported in Table 1. Most of the studies were conducted in the field of oncology/hematology (19), genetic diseases (17) and immunology (7), which represent the three main areas of interest for rare diseases. Two thirds of the conditions can be classified as very rare, with a prevalence lower than 1/100,000 inhabitants, and 29% refer to ultra-rare diseases affecting less than one in a million people.

**Table 1:** Characteristics of the published and unpublished studies included in the analysis (*n* = 62)

	Published		Unpublished		p(chi-square)	Total	
	N	%	N	%		N	%
Studies funded and started	39	62.9	23	37.1		62	100.0
Clinical area					0.604		
Oncology/hematology	11	28.2	8	34.8		19	30.6
Congenital and genetic disorders	13	33.3	4	17.4		17	27.4
Immune system disorders	4	10.3	3	13.0		7	11.3
Other	11	28.2	8	34.8		19	30.6
Prevalence of rare disease					0.381		
> 1/10,000	14	35.9	7	30.4		21	33.9
1–9/100,000	12	30.8	11	47.8		23	37.1
1–9/1,000,000	13	33.3	5	21.8		18	29.0
Study characteristics							
Design					0.055		
Randomized clinical trials (RCT)	21	53.8	18	78.3		39	62.9
Uncontrolled clinical trials (CT)	18	46.2	5	21.7		23	37.1
Type of control group (*n* = 39)	21	100.0	18	100.0	0.584	39	100.0
Active control	11	52.4	11	61.1		22	56.4
Placebo control/no treatment	10	47.6	7	38.9		17	43.6
Planned sample size (tertiles)					0.901		
< =46 patients	13	33.3	8	34.8		21	33.9
47-100 patients	14	35.9	7	30.4		21	33.9
> 100 patients	12	30.8	8	34.8		20	32.3
Mean	98		107			101	
Median	60		80			60	
Q1-Q3	30–120		29–177			30–124	
Special populations					0.307		
Only pediatrics	12	30.8	6	26.1		18	29.0
Also pediatrics	4	10.3	0	0.0		4	6.5
Only adults	15	38.5	9	39.1		24	38.7
Adults and elderly	8	20.5	8	34.8		16	25.8

With regard to study design, 39 projects (63 %) were randomized clinical trials (RCT): in 22 (35.5 %) the comparison included an active treatment, whereas the control group was placebo or no treatment in 17 cases (27.4 %). The median sample size of subjects enrolled in the studies was 60 patients (IQR: 30–124). The patients’ populations covered all age groups, with 18 of the 62 studies (29 %) specifically devoted to the pediatric population.

No statistically significant difference was observed between published and unpublished studies with regard to clinical area, prevalence of the disease, type of control group, sample size, and special population involved. We found a higher proportion of RCTs still unpublished even though the difference did not reach statistical significance (*p* = 0.055).

### Time to publication

**Fig. 2:**
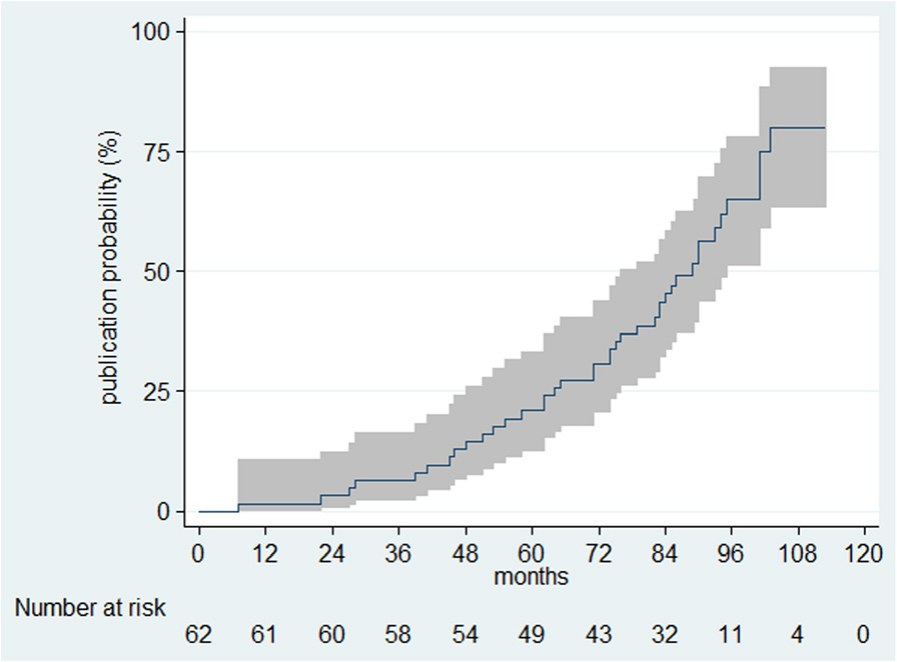
Kaplan-Meier curve of cumulative probability of publication by time (months) since funding agreement and 95 % confidence intervals in the analyzed cohort of studies

The median time from signing the contract to publication of the study results was 74 months (IQR 49–84). The cumulative probability of publication reached 77.2 % at 9 years of follow-up (Fig. 2).

**Fig. 3:**
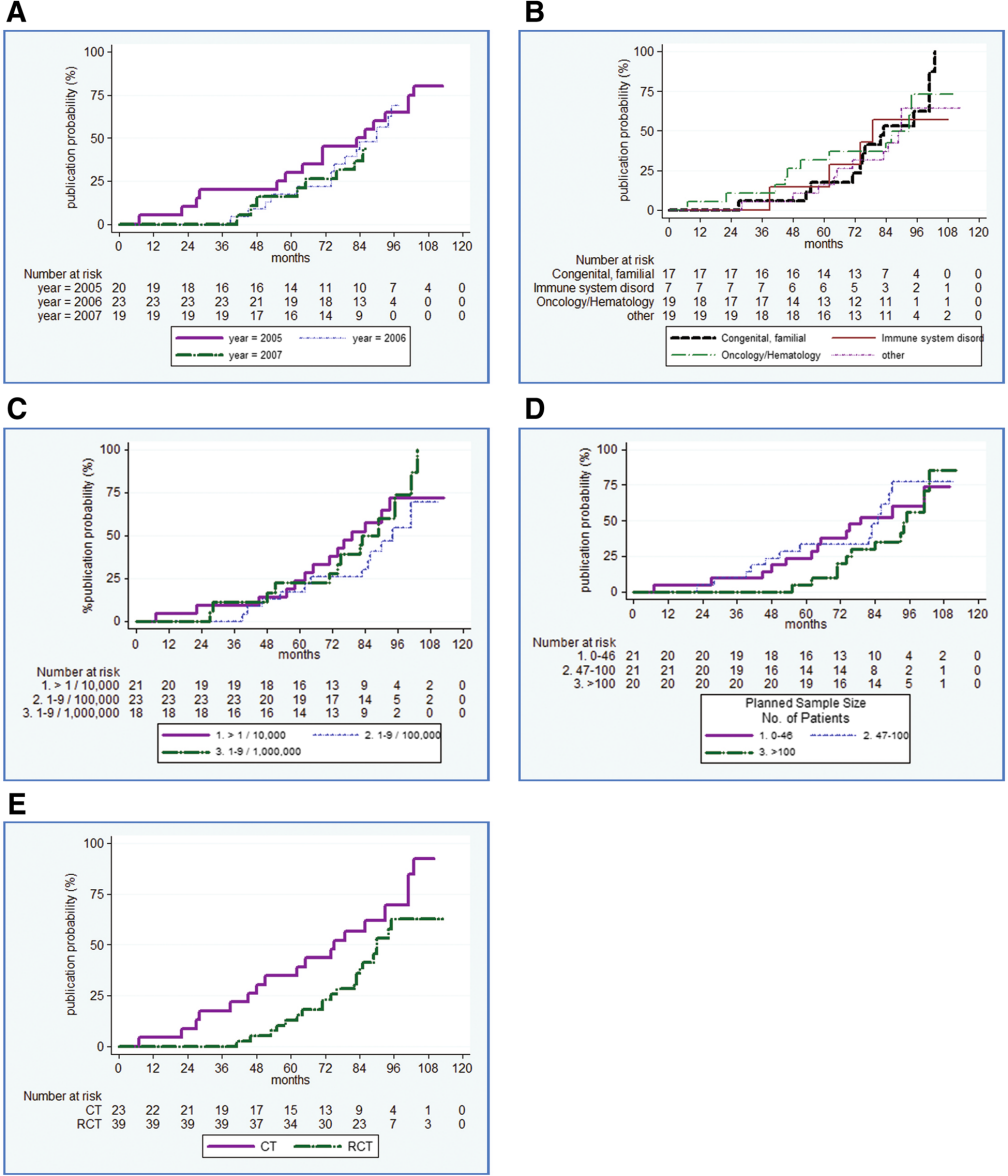
Kaplan–Meier Plot of cumulative probability of publication by time (months) since funding agreement in the analyzed cohort by study characteristics: Year, *p* = 0.8184 (Panel **a**); Clinical area, *p* = 0.9112 (Panel **b**); Disease prevalence, *p* = 0.3759 (Panel **c**); Planned sample size, *p* = 0.3989 (Panel **d**); Study design, i.e. CT vs RCT, *p* = 0.0531 (Panel **e**)

The year of the call (Fig. 3a), the clinical area (Fig. 3b), the prevalence of the disease (Fig. 3c) and the sample size (Fig. 3d) did not influence the probability of publication. Given the differential length of follow-up, 80 % (16/20) of the studies funded in 2005 vs 42 % (8/19) of those funded in 2007 were published by November 2015. Although the cumulative probability of publication over the 9 year period did not differ between study designs, the presence of a randomized allocation of subjects was associated with a longer median time to publication (Fig. 3e).

Restricting the analysis to published articles, we observed that the robustness of the outcome (clinical vs surrogate endpoints) and the IF of the journal were not predictive factors of the time to publication (Additional file 2: Figure S1 and Additional file 3: Figure S2). Only the presence of a control group receiving an active treatment was associated with a slightly longer time to publication (*p* = 0.047) (Additional file 4: Figure S3). We also found a high consistency between the planned sample size (as reported at the beginning of the study in the abstract books) and the final number of subjects reported in the published article (median difference 5 subjects; Additional file 5: Figure S4).

### Relevance of publications and implication of findings for clinical practice

Overall, the 39 published articles totaled 401 points of IF (mean: 10.4; median: 5.4, IQR: 3.6-8.7).

Four articles (10 % of the published studies) had findings that might represent a potential breakthrough in clinical practice (Table 2). Twelve articles presented convincing findings as either conclusive-positive (6/39; 15 %) or conclusive-negative (6/39; 15 %); the remaining articles (23; 59 %) provided the proof of concept regarding new treatment hypotheses and consequently required to be confirmed in further robust clinical studies (examples of studies in these categories are presented in the Additional file 6: Table S2).

**Table 2:** Studies with potentially breakthrough findings

• The project coordinated by Tiziano Barbui was aimed at comparing, in patients with polycythemia vera, two therapeutic strategies (based on pharmacological and non-pharmacological interventions) in the prevention of thrombotic events. In a RCT that included 365 patients, the strategy aimed at maintaining the hematocrit target at less than 45 % (aggressive strategy) was associated with a significantly lower rate of cardiovascular death and major thrombosis in comparison with the group of patients with a hematocrit target of 45 to 50 % (hazard ratio in the high-hematocrit group, 3.91; 95 % CI, 1.45 to 10.53) [25].
• In patients with acute myeloid leukaemia, the standard myeloablative conditioning treatment of busulphan plus cyclophosphamide is associated with a substantial non-relapse mortality. An alternative combination of busulfan and fludarabine has been proposed to reduce the incidence of these events. In a multicenter study, Rambaldi and coll. randomized 252 patients (aged 40-65 years) with acute myeloid leukaemia to compare the two regimens [26]. The 1-year non-relapse mortality was 17.2 % in the busulfan plus cyclophosphamide group and 7.9 % in the busulfan plus fludarabine group (Gray's test p=0.026). No difference was observed in terms of serious adverse events. The Authors concluded that, in older patients with acute myeloid leukemia, the busulfan plus fludarabine regimen “should be regarded as standard of care during the planning of allogeneic transplants”.
• This example refers to a multicenter, randomized, study that compared plasma-derived (PD) with recombinant (R) factor VIII with regard to the risk of developing autoantibodies that neutralize the coagulant activity of factor VIII [19]. Between 2010 and 2014, 251 previously untreated patients with severe hemophilia A were randomized to the recombinant or the plasma-derived products. The cumulative incidence of developing factor VIII inhibitors was higher in the recombinant than in the plasma-derived group (hazard ratio 1.87; 95 % CI 1.18-2.97). These findings are of special importance also for low- and middle-income countries when considering that the plasma-derived factor VIII is far less expensive than the recombinant product.
• In this study, AIFA co-founded a project (already supported by Telethon charity) with the aim to extend the number of patients receiving a gene therapy for the treatment of an extremely rare and severe congenital immunodeficiency due to adenosine deaminase deficiency (ADA) [27]. Aiuti and coll. were able to demonstrate that the treatment could radically modify the prognosis of these patients. After 4 years of follow up, all ten patients were alive and for 8 of them the enzyme-replacement therapy was not required. The findings of the study originated an agreement between Telethon and a pharmaceutical company for the developing and marketing of an “industrialized” product and the recognition of an orphan drug designation by EMA http://www.pharmafile.com/news/396244/gsk-files-ultra-rare-disease-application-ema.

We also attempted to investigate if specific variables (IF, sample size, robustness of outcome, randomization, and treatment in the control group) were associated to reaching conclusive findings (with immediate application in clinical practice) in comparison to the “proof of concept” studies. Although the sample of the published articles was small, resulting in wide confidence intervals, we observed that a randomized design, robust endpoints and high IF were associated with more clinically relevant studies (Additional file 7: Table S3).

At least in one case the study results were explicitly translated into a regulatory decision. On the basis of the study findings, which was aimed at evaluating the efficacy of low-dose rituximab in patients with refractory HCV-associated mixed cryoglobulinemia, AIFA decided that the tested posology could be provided by the NHS to all patients with the condition under study [12].

## Discussion

Assessing the outcomes of a funding program on independent research on drugs requires answering three main questions: 1) were the studies concluded and their findings published? 2) were the results potentially relevant for clinical practice and regulatory decisions? and 3) given the overall outcomes, is it possible to substantiate the specific role of independent research (or, essentially, would any investment in research attain similar results)? Our study suggests that a positive answer can be given to all these issues.

### Likelihood of concluding the studies and publishing the results

During a median observation of 74 months almost two thirds of the funded projects had an article published in an indexed journal. The cumulative probability of publication reached 77.2 % at the maximum observation time (9 years). Our publication rate is in the upper range of what has been documented in analogous surveys. Kasenda and coll., carried out a retrospective cohort on RCTs that were approved by 6 research ethics committees in Switzerland, Germany and Canada between 2000 and 2003 [13]. Out of 894 RCTs involving patients, 530 (59.3 %) had a full publication within a median follow-up of 11.6 years. Moreover, 4.9 % of the trials approved by the ethics committees never started.

A systematic review carried out by the Cochrane collaboration found that only 52.6 % of the study findings that were presented as abstracts at scientific meetings were published in a full article within the 9 years following the abstract presentation [14]. The probability of publication in full was slightly higher for randomized trials (63.1 %).

Other studies focused on the publication rate of the projects that were recorded in ClinicalTrials.gov. In a cross-sectional analysis concerning 10 % of trials that had been registered in CTG after December 1999 and updated as being completed by June 2007, less than half (311/677, 46 %) were published [15]. In a subsequent analysis, Ross and coll. reviewed the pattern of publication of clinical trials that were funded by the National Institutes of Health and registered in CTG [16]. Focusing the analysis on clinical trials that were updated in CTG as having been completed by December 2008, 68 % (249/635) were published in a peer reviewed biomedical journal whereas 32 % remained unpublished after a median follow-up of 51 months from trial completion. Both surveys refer to registered trials that were updated as being completed, and thus the denominator does not take into account the studies that were recorded in CTG but did not update their profile mainly because of early termination. A greater publication rate observed in the AIFA program might be explained by a specific point in the contract, according to which the researchers were committed to disseminate research findings through the publication on peer reviewed journals.

In early 2016, an analysis on rare disease trials registered in CTG showed that less than half of the studies (47 %) had results published in scientific journals within 3 years after the conclusion [17]; moreover, only 35 % of the studies had the findings reported at the CTG website, despite the requirements of the US Food and Drug Administration to posting results within 1 year after trial completion.

### Relevance of publications and implications for clinical practice and regulatory decisions

Focusing on the impact factor of the published articles is are producible approach to assess the relevance of the study findings. Most of the studies funded by AIFA were published in renowned medical journals, with a median IF of 5.4 (and a mean of 10.4 per article). Of note, only 6.6 % (573 out of 8659) of the journals included in the Journal Citation Reports have a 5 year IF higher than 5.38. Moreover, we made a comparison specifically referred to the Italian context. In 2014, the researchers working in the Italian National Institute of Health (Istituto Superiore di Sanità), the largest research institution of the Italian NHS, published 776 articles in indexed journals, with an average IF of 3.87 [18].

When trying to classify the study outcome in terms of relevance for clinical practice, the thought immediately goes to findings that may radically change the prognosis of a disease or condition, to potentially breakthrough therapies, according to the terminology of the Food and Drugs Administration (FDA). Even though at least four studies reported findings that might be included in this category, an important contribution to clinical practice is also provided by confirmative studies, as well as by studies that verified the lack of efficacy of available treatments.

Confirmative studies are especially needed for orphan drugs because the available evidence is often incomplete when these drugs are approved by regulatory agencies. An even lower level of evidence is inevitably accepted when drugs that are marketed for non-rare diseases are suggested, as off-label indications, for rare conditions. It should be considered unethical that, given the lack of sufficient funding of research, treatments that are not adequately tested in clinical studies continue to be suggested. In this regard, while studies confirming the beneficial effect of an off-label drug provide a new option for the treatment of rare diseases, negative findings are also highly informative in order to prevent useless treatments and to avoid wasting resources.

Independent research programs may also be relevant in terms of economic implications. The findings of the study conducted by Peyvandi et al. on patients with Hemophilia A indicate that the plasma-derived factor VIII has a better benefit-risk profile than the far more expensive recombinant ones [19]. In Italy, in 2015, the overall expenditure for factor VIII was estimated at 200 million euro [20], and 80 % of the use is accounted for by recombinant formulations [21]. Considering that plasma derived factor VIII costs at least 30 % less than the recombinant one, the savings that can be achieved by the Italian National Health Service, in a single year, would be larger than the entire amount of the money allocated for the funding of the 62 studies in the area of rare diseases.

### The specificity of independent research funding programs

The role of independent research is to focus on relevant research questions in areas of limited commercial interest. Rare diseases represent a paradigmatic example. The likelihood of economic return decreases with the decreasing prevalence of the condition. Moreover, the commercial interest is not only inevitably lacking when studying off-patent drugs, but also limited when assessing the role of a patented drug in off-label indications. If the drug is already recommended, no increasing sales may derive when the study results support the efficacy, whereas negative findings would damage the marketing. Finally, and in common with non-rare diseases, carrying out comparative RCTs with an active treatment would normally be regarded a riskier activity.

It is thus not surprising that rare diseases are often considered a difficult area to conduct clinical studies. An analysis based on the CTG database compared interventional clinical trials in rare vs. non-rare diseases by reviewing the main characteristics of 24,088 trials registered between January 2006 and September 2012 [22]. Rare disease trials (2,759; 11.5 % of the total) enrolled fewer participants (median 29 vs 62) and were less likely randomized (35.5 % vs 71.6 %). By comparison, the 62 studies that were funded by AIFA were more similar to the non-rare disease group of trials in terms of participants (median of 60 patients) and proportion of randomized studies (62.9 %).

These data testify that robust study designs can be adopted in the area of rare diseases. They also suggest that independent research is more likely to contribute when the assessment of the end points requires long-term follow-up and, predictably, longer time intervals before conclusion. For instance, in an RCT aimed at assessing the efficacy of adjuvant mitotane in prolonging recurrence-free survival in patients with adrenocortical carcinoma at low-intermediate risk of recurrence after complete resection, the website of the project indicated that the conclusion was expected by the end of 2015 (https://clinicaltrials.gov/ct2/show/NCT00777244). This project was funded to test in a randomized study the promising results of an observational study [23]. By design, the duration of the study had an expected minimum duration of 6 years: 4 years for the enrollment period and 2 years of follow-up. Taking into account the enormous amount of work that is devoted to conduct long-term studies, it is likely that relevant publications will follow.

### Limitations of the study

We cannot exclude that, despite the extensive search that was carried out, we may have missed some of the study publications, which would underestimate the success rate. This is probably the case since after the end of the study follow up (November 2015) the results of a trial funded in 2006 in the setting of juvenile dermatomyositis (demonstrating that prednisone and either ciclosporin or methotrexate was more effective than prednisone alone) were published in the Lancet [24]. Moreover, even considering a long-term follow-up (with a maximum duration of 109 months for the 2005 call) there is an underrepresentation of the publications relevant to the 2007 (and partially 2006) projects. However, the time to publication is similar for the 3 years and we do not expect any change in the final publication rate.

As for the studies that were terminated before completion, no public information was available concerning the reasons for the interruption. In any case, stopping a study did not entail wasting public resources. By contract, funding was transferred to the researcher’s institution only after having verified that specific milestones were reached. In addition, in case of interruption, the researcher’s institution was required to refund the resources that were not already allocated.

## Conclusions

The AIFA program ongoing between 2005 and 2007 in Italy in the area of rare diseases can be considered a success in terms of concluded studies, with a cumulative probability of publication that reached almost 80 %. Our data suggest that adequately powered randomized trials can represent the gold standard also for rare diseases. There are also important implications for clinical practice, as can be expected by projects characterized by clinical endpoints and extensive follow-up. It is unlikely that many of these studies may have been conducted in a for profit perspective, given the absence of commercial interest.

It is unfortunate that the Italian program for independent research on drugs in the area of rare diseases was only active for 3 years [8]. It is possible that the absence of short term results may have triggered a shortsighted decision. We documented that, even though it takes time to set up and conduct a funding program for independent research, the overall results are highly rewarding. Independent funding is crucial in supporting studies aimed at answering questions that are relevant for clinical practice despite the lack of sufficient commercial interest.

### Ethics approval

All data used in the present paper derive from publicly available documents; the analyses only refer to funded projects and published articles. No ethical approval was required.

### Availability of data and materials

The dataset supporting the conclusions of this article is included within the article (and its additional files). No additional data are available. The authors are willing to collaborate in answering further research questions and to participate in systematic reviews or meta-analyses.

## Additional files

Additional file 1: Table S1. Reference list of the 39 published studies in the area rare diseases. (DOC 126 kb) Additional file 2: Figures S1. Kaplan-Meier curve of cumulative probability of publication by time (months) since funding agreement by robustness of outcome in the sub-group of published studies (*p* = 0.5862). (DOCX 36 kb) Additional file 3: Figures S2. Kaplan-Meier curve of cumulative probability of publication by time (months) since funding agreement by impact factor of the journal in the sub-group of published studies (*p* = 0.0849). (DOCX 35 kb) Additional file 4: Figure S3. Kaplan-Meier curve of cumulative probability of publication by time (months) since funding agreement by type of control group in the sub-group of published studies (*p* = 0.0469). (DOCX 36 kb) Additional file 5: Figure S4. Difference between planned and real sample size among published studies (*n* = 39). (PPTX 143 kb) Additional file 6: Table S2. Examples of implications of the study findings. (DOCX 27 kb) Additional file 7: Table S3. Association of selected variables and clinical relevance of the published studies (*n* = 39). (DOCX 21 kb)

## References

[1] Chalmers I, Glasziou P (2009). Avoidable waste in the production and reporting of research evidence. Lancet.

[2] Macleod MR, Michie S, Roberts I (2014). Biomedical research: increasing value, reducing waste. Lancet.

[3] Liberati A (2004). An unfinished trip through uncertainties. BMJ.

[4] Liberati A (2011). Need to realign patient-oriented and commercial and academic research. Lancet.

[5] World Medical Association (2013). Declaration of Helsinki: ethical principles for medical research involving human subjects. JAMA.

[6] Italian Medicines Agency (AIFA) Research & Development Working Group (2010). Feasibility and challenges of independent research on drugs: the Italian Medicines Agency experience. Eur J Clin Invest.

[7] Ufficio Ricerca e Sviluppo e Commissione Ricerca e Sviluppo (2008). Rapporto sull’organizzazione della ricerca indipendente sui farmaci promossa dall’AIFA nel triennio 2005–2007.

[8] Traversa G. I ritardi della ricerca indipendente Aifa. Ricerca e Pratica 2014;30:270–2. http://www.ricercaepratica.it/articoli.php?archivio=yes&vol_id=1714&id=18714. Accessed 28 Feb 2016.

[9] Ufficio Ricerca e Sviluppo AIFA (2008). La ricerca indipendente sui farmaci promossa dall’AIFA: Riassunti degli studi finanziati all’interno dei bandi AIFA 2005 e 2006.

[10] Ufficio Ricerca e Sviluppo AIFA (2009). La ricerca indipendente sui farmaci promossa dall’AIFA: Riassunti degli studi finanziati all’interno dei bandi AIFA 2005-2006-2007.

[11] 2015 Journal Citation Reports®.(Thomson Reuters, 2015)

[12] Visentini M, Ludovisi S, Petrarca A (2011). A phase II, single-arm multicenter study of low-dose rituximab for refractory mixed cryoglobulinemia secondary to hepatitis C virus infection. Autoimmun Rev.

[13] Kasenda B, von Elm E, You J (2014). Prevalence, characteristics, and publication of discontinued randomized trials. JAMA.

[14] Scherer RW, Langenberg P, von Elm E (2007). Full publication of results initially presented in abstracts. Cochrane Database Syst Rev.

[15] Ross JS, Mulvey GK, Hines EM (2009). Trial publication after registration in clinicaltrials.gov: A cross-sectional analysis. PLoS Med.

[16] Ross JS, Tse T, Zarin DA (2011). Publication of NIH funded trials registered in clinicaltrials.gov: cross sectional analysis. BMJ.

[17] Dechartres A, Riveros C, Harroch M, Faber T, Ravaud P (2016). Characteristics and public availability of results of clinical trials on rare diseases registered at clinicaltrials.gov. JAMA Intern Med.

[18] Istituto Superiore di Sanità (2015). Relazione dell’Istituto Superiore di Sanità sui risultati dell’attività svolta nel 2014.

[19] Peyvandi F, Mannucci PM, Garagiola I (2015). Source of factor VIII replacement (plasmatic or recombinant) and incidence of inhibitory alloantibodies in previously untreated patients with severe hemophilia a: The multicenter randomized sippet study.

[20] The Medicines Utilization Monitoring Centre (2016). National report on medicines use in italy. January-September 2015.

[21] Calizzani G, Lanzoni M, Candura F (2012). Analisi della domanda dei principali medicinali plasmaderivati in Italia. Anni 2007–2011.

[22] Bell SA, Tudur-Smith C (2014). A comparison of interventional clinical trials in rare versus non-rare diseases: an analysis of ClinicalTrials.gov. OJRD.

[23] Terzolo M, Angeli A, Fassnacht M (2007). Adjuvant mitotane treatment for adrenocortical carcinoma. N Engl J Med.

[24] Ruperto N, Pistorio A, Oliverira S (2016). Prednisone versus prednisone plus ciclosporin versus prednisone plus methotrexate in new-onset juvenile dermatomyositis: a randomised trial. Lancet.

[25] Marchioli R, Finazzi G, Specchia G (2013). Cardiovascular events and intensity of treatment in polycythemia vera. N Engl J Med.

[26] Rambaldi A, Grassi A, Masciulli A (2015). Busulfan plus cyclophosphamide versus busulfan plus fludarabine as a preparative regimen for allogeneic haemopoietic stem-cell transplantation in patients with acute myeloid leukaemia: an open-label, multicentre, randomised, phase 3 trial. Lancet Oncol.

[27] Aiuti A, Cattaneo F, Galimberti S (2009). Gene therapy for immunodeficiency due to adenosine deaminase deficiency. N Engl J Med.

