# Supplementary material for: Italian program for independent research on drugs: 10 year follow-up of funded studies in the area of rare diseases
Source: Orphanet J Rare Dis. 2016 Apr 12;11:36. doi: 10.1186/s13023-016-0420-4 (PMC4828875; doi:10.1186/s13023-016-0420-4)
Supplement: Additional file 1: Table S1. — Reference list of the 39 published studies in the area rare diseases. (DOC 126 kb) [file 13023_2016_420_MOESM1_ESM.doc]

**Additional file 1: Table S1. Reference list of the 39 published studies in the area rare diseases.**

| **N°** | **Principal Investigator** | **Call**  **(Year)** | **Study title** | **Publication reference** |
| --- | --- | --- | --- | --- |
| 1 | **Abbate Mauro** | **2007** | A prospective, sequential study to assess the efficacy of rituximab therapy in maintaining remission of nephrotic syndrome after steroid and immunosuppressive therapy withdrawal in patients with steroid-dependant or multirelapsing minimal change disease or focal segmental glomerulosclerosis | Ruggenenti P, Ruggiero B, Cravedi P, et al. Rituximab in Nephrotic Syndrome of Steroid-Dependent or Frequently Relapsing Minimal Change Disease Or Focal Segmental Glomerulosclerosis (NEMO) Study Group. Rituximab in steroid-dependent or frequently relapsing idiopathic nephrotic syndrome. J Am Soc Nephrol 2014;25:850-63. |
| 2 | **Aiuti Alessandro** | **2005** | Safety and efficacy study of gene therapy with autologous CD34+ cells transfected with a retroviral vector encoding the adenosine deaminase (ADA) gene (EMEA/OD/053/05) for the treatment of severe combined immunodeficiency due to the lack of ADA | Aiuti A, Cattaneo F, Galimberti S, et al. Gene therapy for immunodeficiency due to adenosine deaminase deficiency. N Engl J Med 2009;360:447-58. |
| 3 | **Andria Generoso** | **2005** | Efficacy and safety of treatment with N-butyl-deoxynojirimycin (NB-DNJ-miglustat) in patients with Niemann-Pick disease type C | Fecarotta S, Romano A, Della Casa R, et al. Long term follow-up to evaluate the efficacy of miglustat treatment in Italian patients with Niemann-Pick disease type C. Orphanet J Rare Dis 2015;10:22. |
| 4 | **Andria Generoso** | **2005** | Multicentre study of the efficacy and tolerability of tetrahydrobiopterin in a pediatric population affected by phenylketonuria | Scala I, Concolino D, Della Casa R, et al. Long-term follow-up of patients with phenylketonuria treated with tetrahydrobiopterin: a seven years experience. Orphanet J Rare Dis 2015;10:14. |
| 5 | **Ardizzoni Andrea** | **2006** | Multicentre phase III randomized study of cisplatin and etoposide with or without bevacizumab as first-line treatment in extensive stage (ED) small cell lung cancer (SCLC) | Tiseo M, Boni L, Ambrosio F, et al. Italian multicenter phase III randomized study of cisplatin-etoposide with or without bevacizumab as first-line treatment in extensive stage small cell lung cancer: treatment rationale and protocol design of the GOIRC-AIFA FARM6PMFJM trial. Clin Lung Cancer 2015;16:67-70. |
| 6 | **Barbui Tiziano** | **2006** | Intensity of cyto-reductive therapy to prevent cardiovascular events in patients with Polycythemia vera (PV) – CYTO-PV | Marchioli R, Finazzi G, Specchia G, et al. Cardiovascular events and intensity of treatment in polycythemia vera. N Engl J Med 2013;368:22-33. |
| 7 | **Beghi Ettore** | **2005** | Double-blind placebo-controlled trial on the use of acetyl-L carnitine for the treatment of amyotrophic lateral sclerosis (ALS) | Beghi E, Pupillo E, Bonito V, et al. Randomized double-blind placebo-controlled trial of acetyl-L-carnitine for ALS. Amyotroph Lateral Scler Frontotemporal Degener 2013;14:397-405. |
| 8 | **Berni Canani Roberto** | **2006** | Therapeutic efficacy of butyrate in pediatric patients with congenital chloride diarrhea | Canani RB, Terrin G, Elce A, et al. Genotype-dependency of butyrate efficacy in children with congenital chloride diarrhea. Orphanet J Rare Dis 2013;8:194. |
| 9 | **Calabrò Raffaele** | **2006** | Arterial hypertension after successful aortic decoarctation: atenolol vs enalapril comparison of efficacy and tolerability in pediatric age | Di Salvo G, Castaldi B, Gala S, et al. Atenolol vs enalapril in young hypertensive patients after successful repair of aortic coarctation. J Hum Hypertens 2015 doi:10.1038/jhh.2015.87 |
| 10 | **Casali Paolo Giovanni** | **2005** | Trabectedin (ET743) in metastatic or locally advanced myxoid/round cell liposarcoma pretreated with chemotherapy | Grosso F, Sanfilippo R, Virdis, E et al. Trabectedin in myxoid liposarcomas (MLS): a long-term analysis of a single-institution series. Ann Oncol 2009;20:1439-44. |
| 11 | **Cavagnini Francesco** | **2007** | Evaluation of the benefit-risk profile of retinoic acid in the treatment of Cushing's disease | Pecori Giraldi F, Ambrogio AG, Andrioli M, et al. Potential role for retinoic acid in patients with Cushing's disease. J Clin Endocrinol Metab 2012;97:3577-83. |
| 12 | **Colao Annamaria** | **2005** | Effectiveness and tolerability of treatment with cabergoline in Cushing's syndrome | Pivonello R, De Martino MC, Cappabianca P, et al. The medical treatment of Cushing's disease: effectiveness of chronic treatment with the dopamine agonist cabergoline in patients unsuccessfully treated by surgery. J Clin Endocrinol Metab 2009;94:223-30. |
| 13 | **Corradini Paolo** | **2005** | Intensive chemo-immunotherapy as first-line treatment in adult patients with peripheral T-cell Lymphoma (PTCL) | Corradini P, Vitolo U, Rambaldi A, et al. Intensified chemo-immunotherapy with or without stem cell transplantation in newly diagnosed patients with peripheral T-cell lymphoma. Leukemia 2014;28:1885-91. |
| 14 | **De Santo Natale Gaspare** | **2005** | Therapy of hyperhomocysteinemia in hemodialysis patients: effects of acetylcysteine and folates | Perna AF, [Violetti E](http://www.ncbi.nlm.nih.gov/pubmed/?term=Violetti E%5BAuthor%5D&cauthor=true&cauthor_uid=22226754), [Lanza D](http://www.ncbi.nlm.nih.gov/pubmed/?term=Lanza D%5BAuthor%5D&cauthor=true&cauthor_uid=22226754), et al. Therapy of hyperhomocysteinemia in hemodialysis patients: effects of folates and N-acetylcysteine. J Ren Nutr 2012;22:507-514.e1 doi:10.1053/j.jrn.2011.10.007. |
| 15 | **Fais Stefano** | **2005** | Phase II clinical study on efficacy of proton pump inhibitors pre-treatment in osteosarcoma patients undergoing chemotherapy | Ferrari S, Perut F, Fagioli F, et al. Proton pump inhibitor chemosensitization in human osteosarcoma: from the bench to the patients' bed. J Transl Med 2013;11:268. |
| 16 | **Filla Alessandro** | **2005** | Growth hormone in patients with amyotrophic lateral sclerosis as add-on therapy to Riluzole | Saccà F, Quarantelli M, Rinaldi C, et al. A randomized controlled clinical trial of growth hormone in amyotrophic lateral sclerosis: clinical, neuroimaging, and hormonal results. J Neurol 2012;259:132-8. |
| 17 | **Fiorilli Massimo** | **2006** | Evaluation of the benefit/cost/safety profile of low-dose anti-CD20 monoclonal antibody (rituximab) treatment for refractory mixed cryoglobulinemia | Visentini M, Ludovisi S, Petrarca A, et al. A phase II, single-arm multicenter study of low-dose rituximab for refractory mixed cryoglobulinemia secondary to hepatitis C virus infection. Autoimmun Rev 2011;10:714-9. |
| 18 | **Fraticelli Paolo** | **2006** | Low-dose Oral Imatinib in the Treatment of Scleroderma Pulmonary Involvement: a Phase II pilot study | Fraticelli P, Gabrielli B, Pomponio G, et al. Low-dose oral imatinib in the treatment of systemic sclerosis interstitial lung disease unresponsive to cyclophosphamide: a phase II pilot study. Arthritis Res Ther 2014;16:R144 doi:10.1186/ar4606. |
| 19 | **Gringeri Alessandro** | **2007** | Inhibitor development in previously untreated patients (pups) with severe haemophilia A when exposed to von Willebrand factor-containing plasma-derived factor VIII concentrates and to recombinant factor VIII concentrates: an international, multicentre, prospective, controlled, open label, randomised, clinical trial | Peyvandi F, Mannucci PM, Garagiola I, et al. Source of Factor VIII replacement (plasmatic or recombinant) and incidence of inhibitory alloantibodies in previously untreated patients with severe hemophilia A: the multicenter randomized SIPPET study. 57th ASH Annual Meeting & Exposition, Orlando, FL, December 5-8, 2015, <https://ash.confex.com/ash/2015/webprogram/Paper82866.html> (Submitted to Blood) |
| 20 | **Lambiase Alessandro** | **2005** | Multicentre, randomised, double masked, controlled studies on the efficacy of Cyclosporine eye drop treatment in preventing Vernal Keratoconjunctivitis (VKC) relapses and in treating the acute phase | Lambiase A, Leonardi A, Sacchetti M, Deligianni V, Sposato S, Bonini S. Topical cyclosporine prevents seasonal recurrences of vernal keratoconjunctivitis in a randomized, double-masked, controlled 2-year study. J Allergy Clin Immunol 2011;128:896-897.e9. |
| 21 | **Locatelli Franco** | **2005** | Use of anti-CD20 monoclonal antibody (rituximab) for the prevention and/or treatment of EBV-associated lymphoproliferative disease in recipients of hematopoietic stem cell and solid organ transplantation or in patients with primary immunodeficiency | Comoli P, Basso S, Zecca M, et al. Preemptive therapy of EBV-related lymphoproliferative disease after pediatric haploidentical stem cell transplantation. Am J Transplant 2007;7:1648-55. |
| 22 | **Marsico Serafino** | **2007** | Randomized, single blind, controlled trial of inhaled glutathione versus placebo in patients with cystic fibrosis | Calabrese C, Tosco A, Abete P, et al. Randomized, single blind, controlled trial of inhaled glutathione vs placebo in patients with cystic fibrosis. J Cyst Fibros 2015;14:203-10. |
| 23 | **Nobili Bruno** | **2005** | A randomized, open-label therapeutic trial for the evaluation of the efficacy and safety of Neridronate (Nerixia) in the treatment of osteoporosis in patients with Thalassemia Major and Severe Thalassemia Intermedia | Forni GL, Perrotta S, Giusti A, et al. Neridronate improves bone mineral density and reduces back pain in β-thalassaemia patients with osteoporosis: results from a phase 2, randomized, parallel-arm, open-label study. Br J Haematol 2012;158:274-82. |
| 24 | **Olivieri Attilio** | **2007** | Imatinib Mesylate in the treatment of refractory extensive chronic Graft Versus Host Disease (cGVHD) with features Scleroderma-like | Olivieri A, Cimminiello M, Corradini P, et al. Long-term outcome and prospective validation of NIH response criteria in 39 patients receiving imatinib for steroid-refractory chronic GVHD. Blood 2013;122:4111-8. |
| 25 | **Palmieri Giovannella** | **2006** | Phase II monitored clinical trial for evaluation of treatment of patients with Thymic Epithelial Tumours (TET) or Histiocytosis X (LCH) with Imatinib Mesylate | Palmieri G, Marino M, Buonerba C, et al. Imatinib mesylate in thymic epithelial malignancies. Cancer Chemother Pharmacol 2012;69:309-15. |
| 26 | **Parenti Giancarlo** | **2005** | Evaluation of the efficacy of the treatment with ACE-inhibitors on the renal damage in patients affected by glycogen storage disease type 1a and type 1b and of the vitamin E on neutropenia of patients with glycogen storage disease 1b | Melis D, Cozzolino M, Minopoli G, et al. Progression of renal damage in glycogen storage disease type I is associated to hyperlipidemia: a multicenter prospective Italian study. J Pediatr 2015;166:1079-82. |
| 27 | **Pareyson Davide** | **2005** | Multicentre randomised double blind placebo controlled trial of long-term ascorbic acid treatment in Charcot-Marie-Tooth disease type 1a (CMT-trial: CMT-trial Italian with ascorbic acid long term) | Pareyson D, Reilly MM, Schenone A, et al. Ascorbic acid in Charcot-Marie-Tooth disease type 1A (CMT-TRIAAL and CMT-TRAUK): a double-blind randomised trial. Lancet Neurol 2011;10:320-8. |
| 28 | **Parodi Oberdan** | **2006** | Effects of tetrahydrobiopterin (BH4) on flow-mediated dilation in Cadasil patients: a randomised controlled trial | De Maria R, Campolo J, Frontali M, et al. Effects of sapropterin on endothelium-dependent vasodilation in patients with CADASIL: a randomized controlled trial. Stroke 2014;45:2959-66. |
| 29 | **Pucci Neri** | **2006** | Multicentrici comparative randomized double blind cross over study with tacrolimus (FK506) 0.1% eyedrops and cyclosporine 1% eyedrops in children with severe active vernal keratoconjunctivitis, lasting 7 weeks, followed by an open trial lasting 24 months to evaluate long term efficacy and safety of the treatment | Pucci N, Caputo R, di Grande L, et al. Tacrolimus vs. cyclosporine eyedrops in severe cyclosporine-resistant vernal keratoconjunctivitis: A randomized, comparative, double-blind, crossover study. Pediatr Allergy Immunol 2015;26:256-61. |
| 30 | **Rambaldi Alessandro** | **2006** | Randomized study comparing intravenous busulfan (i.v. bu; busilvex®) plus cyclophosphamide (bucy2) versus intravenous busulfan plus fludarabine (buflu) as conditioning regimens prior to allogenic hematopoietic stem cell transplantation in patients with acute myeloid leukemia, chronic myeloid leukemia and myelodysplastic syndrome | Rambaldi A, Grassi A, Masciulli A, et al. Busulfan plus cyclophosphamide versus busulfan plus fludarabine as a preparative regimen for allogeneic haemopoietic stem-cell transplantation in patients with acute myeloid leukaemia: an open-label, multicentre, randomised, phase 3 trial. Lancet Oncol 2015;16:1525-36. |
| 31 | **Redaelli Tiziana** | **2006** | Evaluation of the tolerability and efficacy of erythropoietin (EPO) treatment in spinal shock: comparative study VS methylprednisolone | Costa DD, Beghi E, Carignano P, et al. Tolerability and efficacy of erythropoietin (EPO) treatment in traumatic spinal cord injury: a preliminary randomized comparative trial vs. methylprednisolone (MP). Neurol Sci 2015;36:1567-74. |
| 32 | **Ristori Giovanni** | **2007** | Efficacy of riluzole in hereditary cerebellar ataxia: a randomized double-blind placebo-controlled trial | Romano S, Coarelli G, Marcotulli C, et al. Riluzole in patients with hereditary cerebellar ataxia: a randomised, double-blind, placebo-controlled trial. Lancet Neurol 2015;14:985-91. |
| 33 | **Sansonno Domenico** | **2007** | Use of sorafenib in the prevention of relapse of resected/ablated hepatocellular carcinoma in patients with hepatitis C virus chronic infection. a phase II randomized, controlled trial | Sansonno D, Lauletta G, Russi S, Conteduca V, Sansonno L, Dammacco F. Transarterial chemoembolization plus sorafenib: a sequential therapeutic scheme for HCV-related intermediate-stage hepatocellular carcinoma: a randomized clinical trial. Oncologist 2012;17:359-66. |
| 34 | **Scarpa Maurizio** | **2006** | Evaluation of the efficacy of enzyme replacement therapy in pediatric patients affected by mucopolysaccharidosis type II (Hunter syndrome) | Tomanin R, Zanetti A, D'Avanzo F, et al. Clinical efficacy of enzyme replacement therapy in paediatric Hunter patients, an independent study of 3.5 years. Orphanet J Rare Dis 2014;9:129. |
| 35 | **Spada Anna Maria** | **2007** | Treatment of hyperparathyroidism in patients with multiple endocrine neoplasia type 1 (MEN1) with the calcimimetic agent cinacalcet | Filopanti M, Verga U, Ermetici F, et al. MEN1-related hyperparathyroidism: response to cinacalcet and its relationship with the calcium-sensing receptor gene variant Arg990Gly. Eur J Endocrinol 2012;167:157-64. |
| 36 | **Tagliavini Fabrizio** | **2005** | A randomized, double-blind pilot study versus placebo for the evaluation of the efficacy of doxycycline administered by oral route in patients affected by Creutzfeldt-Jakob disease | Haïk S, Marcon G, Mallet A, et al. Doxycycline in Creutzfeldt-Jakob disease: a phase 2, randomised, double-blind, placebo-controlled trial. Lancet Neurol 2014;13:150-8. |
| 37 | **Taroni Franco** | **2006** | Randomized placebo-controlled double-blind trial to assess safety and efficacy of erythropoietin in adult patients with Friedreich’s ataxia (pilot study) | Mariotti C, Fancellu R, Caldarazzo S, et al. Erythropoietin in Friedreich ataxia: no effect on frataxin in a randomized controlled trial. Mov Disord 2012;27:446-9. |
| 38 | **Vannucchi Alessandro** | **2006** | A phase 2 study of efficacy/safety of everolimus in subjects with idiopathic myelofibrosis (IMF) | Guglielmelli P, Barosi G, Rambaldi A, et al. Safety and efficacy of everolimus, a mTOR inhibitor, as single agent in a phase 1/2 study in patients with myelofibrosis. Blood 2011;118:2069-76. |
| 39 | **Ventura Alessandro** | **2006** | Randomized controlled double-blind vs. placebo multicentre study on the safety and effectiveness of thalidomide in the treatment of refractory Crohn’s disease and ulcerative colitis in children and adolescents | Lazzerini M, Martelossi S, Magazzù G, et al. Effect of thalidomide on clinical remission in children and adolescents with refractory Crohn disease: a randomized clinical trial. JAMA 2013;310:2164-73. |
