# Supplementary material for: Italian program for independent research on drugs: 10 year follow-up of funded studies in the area of rare diseases
Source: Orphanet J Rare Dis. 2016 Apr 12;11:36. doi: 10.1186/s13023-016-0420-4 (PMC4828875; doi:10.1186/s13023-016-0420-4)

Additional file 3: Figure S2. Kaplan-Meier curve of cumulative probability of publication by time (months) since funding agreement by impact factor of the journal in the sub-group of published studies (p = 0.0849).


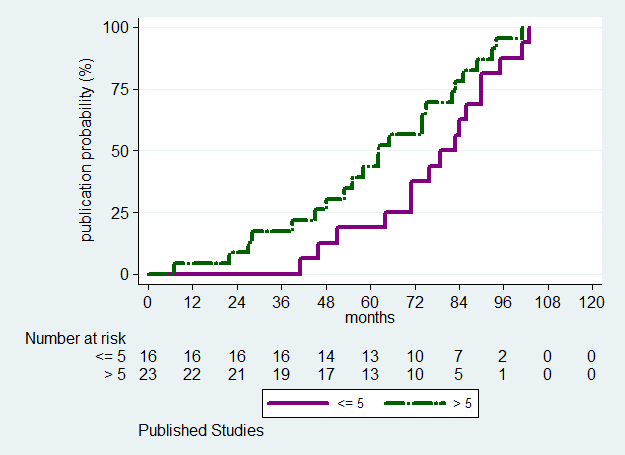

Supplement: Additional file 3: Figures S2. — Kaplan-Meier curve of cumulative probability of publication by time (months) since funding agreement by impact factor of the journal in the sub-group of published studies (p = 0.0849). (DOCX 35 kb) [file 13023_2016_420_MOESM3_ESM.docx]
