# Supplementary material for: Italian program for independent research on drugs: 10 year follow-up of funded studies in the area of rare diseases
Source: Orphanet J Rare Dis. 2016 Apr 12;11:36. doi: 10.1186/s13023-016-0420-4 (PMC4828875; doi:10.1186/s13023-016-0420-4)

Additional file 4: Figure S3. Kaplan-Meier curve of cumulative probability of publication by time (months) since funding agreement by type of control group in the sub-group of published studies (p =0.0469)


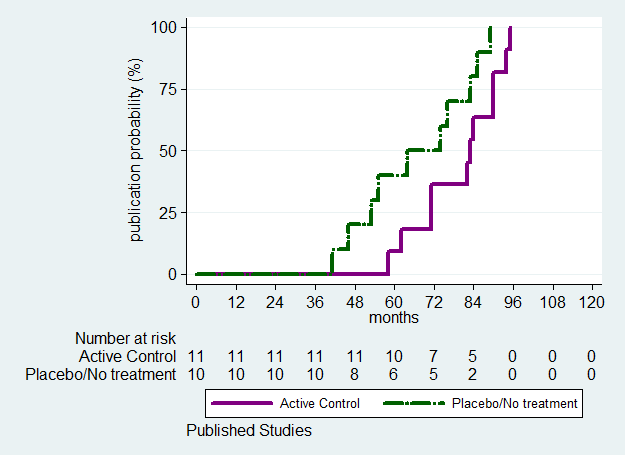

Supplement: Additional file 4: Figure S3. — Kaplan-Meier curve of cumulative probability of publication by time (months) since funding agreement by type of control group in the sub-group of published studies (p = 0.0469). (DOCX 36 kb) [file 13023_2016_420_MOESM4_ESM.docx]
