# Supplementary figures and images for: Italian program for independent research on drugs: 10 year follow-up of funded studies in the area of rare diseases
Source: Orphanet J Rare Dis. 2016 Apr 12;11:36. doi: 10.1186/s13023-016-0420-4 (PMC4828875; doi:10.1186/s13023-016-0420-4)

## Slide 1
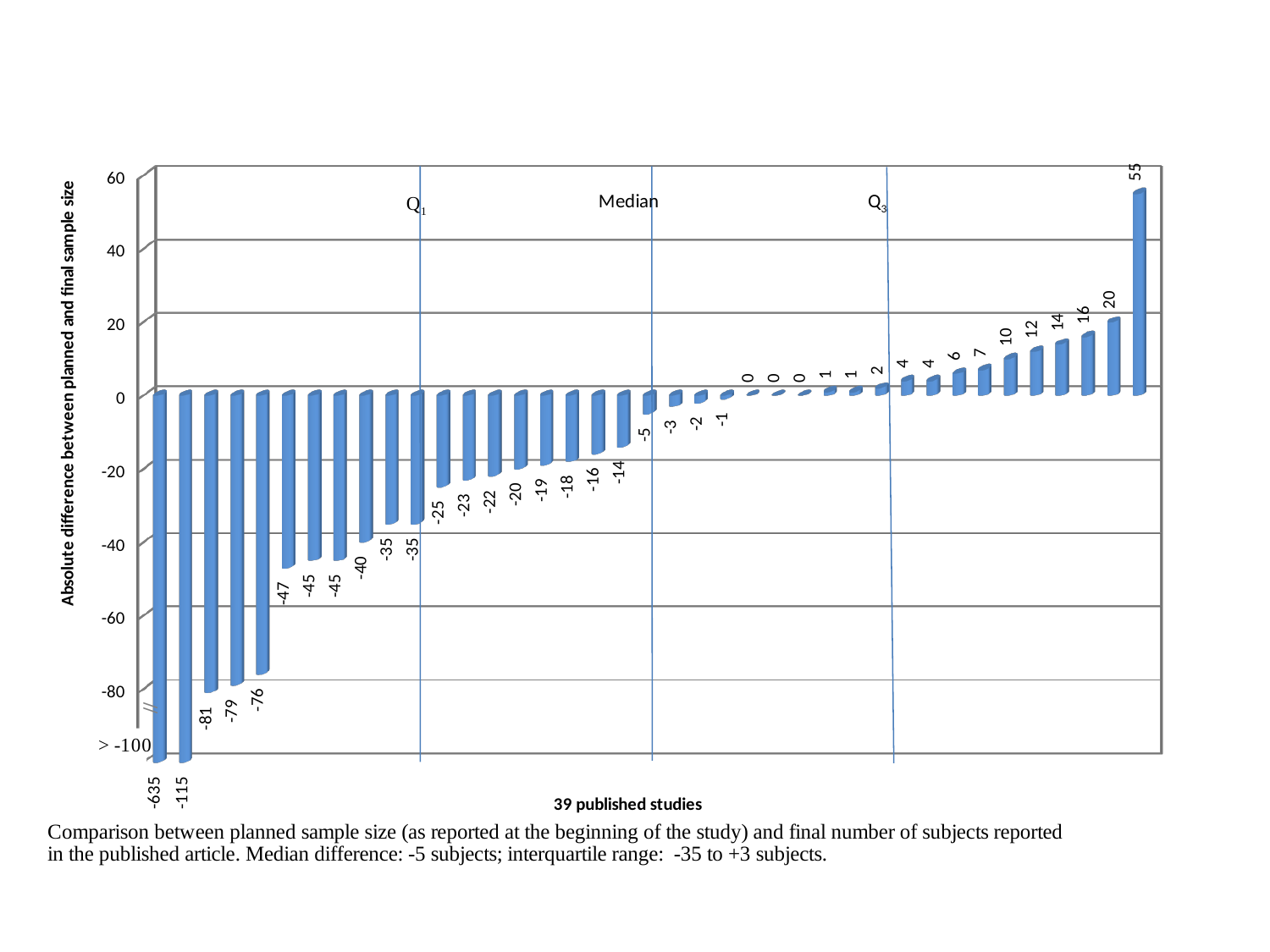

[unsupported chart]

## Slide 2
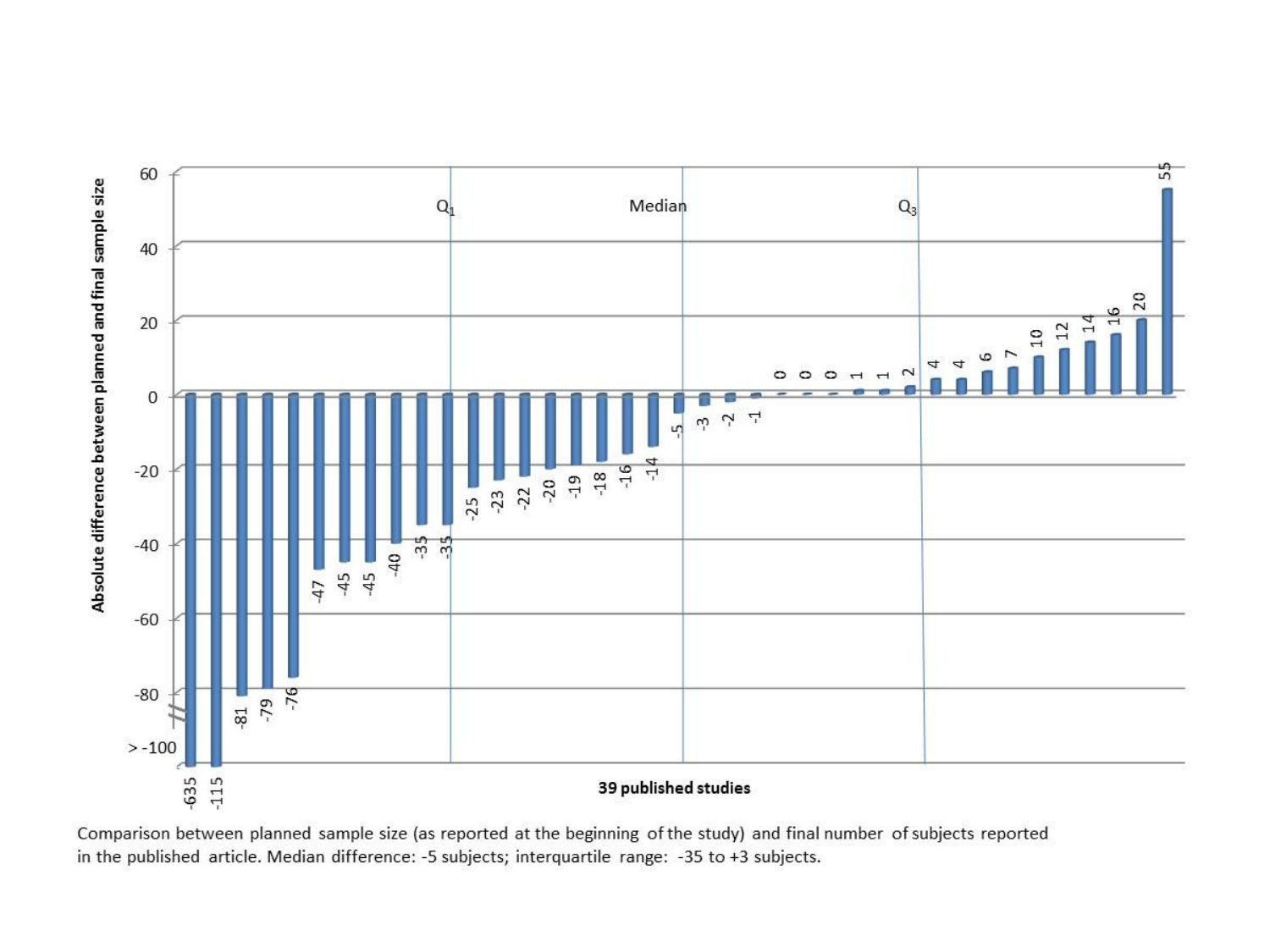

Supplement: Additional file 5: Figure S4. — Difference between planned and real sample size among published studies (n = 39). (PPTX 143 kb) [file 13023_2016_420_MOESM5_ESM.pptx]
