# Supplementary material for: Italian program for independent research on drugs: 10 year follow-up of funded studies in the area of rare diseases
Source: Orphanet J Rare Dis. 2016 Apr 12;11:36. doi: 10.1186/s13023-016-0420-4 (PMC4828875; doi:10.1186/s13023-016-0420-4)
Supplement: Additional file 6: Table S2. — Examples of implications of the study findings. (DOCX 27 kb) [file 13023_2016_420_MOESM6_ESM.docx]

**Additional file 6: Table S2. Examples of implications of the study findingsaccording to the category of clinical relevance** (principal investigator, project title and synthesis of the findings are reportedfor each study)

**Conclusive-positive**

***Fiorilli Massimo - Evaluation of the benefit/cost/safety profile of low-dose anti-CD20 monoclonal antibody (rituximab) treatment for refractory mixed cryoglobulinemia***

In a phase-II, single-arm multicenter study, Fiorilli and coll. aimed at evaluating the efficacy of low-dose rituximab in patients with refractory HCV-associated mixed cryoglobulinemia [1]. Specifically, they intended to verify whether reducing the rituximab dosage to 250 mg/m^2^ given twice at one-week interval might approximate the clinical response that was expected in patients receiving 375 mg/m^2^ given four times (around 80%). The overall response rate in the first 24 evaluable patients was 79%, with a mean time to relapse of 6.5 months (similar to those reported in studies using high-dose rituximab). The study findings informed a regulatory decision: the Italian Medicines Agency decided that the posology tested in the study could be provided by the Italian NHS to all patients with the condition under study (AIFA. Determina 18 aprile 2014. Inserimento del medicinale rituximab nell'elenco dei medicinali erogabili a totale carico del Servizio sanitario nazionale. GU Serie Generale n.103 del 6-5-2014).

***Andria Generoso - Efficacy and safety of treatment with N-butyl-deoxynojirimycin (NB-DNJ-miglustat) in patients with Niemann-Pick disease type C***

In the multicenter study coordinated by Andria, 25 patients with Niemann-Pick disease type C were treated with miglustat for periods ranging from 48 to 96 months [2]. When the project was submitted in the 2005 call, patients with Niemann-Pick were often treated off-label (at the time miglustat was only approved for the treatment of Gaucher syndrome) according to preliminary evidence deriving from an industry sponsored trial. The study findings “suggest that miglustat can improve or stabilize neurological manifestations, at least for a period of time…”. Of note, when in 2009 miglustat was approved by EMA for the treatment of Niemann-Pick, the evidence provided by the marketing authorization holder was still limited and EMA required that further studies be conducted to gain the needed information. To date, the Italian study represents the largest follow up conducted in this patient population.

**Conclusive-negative**

***Pareyson Davide – Multicentre randomised double blind placebo controlled trial of long-term ascorbic acid treatment in Charcot-Marie-Tooth disease type 1a (CMT-trial: CMT-trial Italian with ascorbic acid long term)***

In a multinational double-blind randomised trial, Pareyson and coll. verified the efficacy of ascorbic acid in Charcot-Marie-Tooth disease type 1A (CMT1A) [3]. Animal models had suggested a potential role of this substance in reducing the severity of neuropathy but short term trials had found no benefit in human beings. In a trial with 24 months follow-up, 277 patients were randomly assigned to receive 1.5 g/day oral ascorbic acid or matching placebo. The authors found no differences between the groups in both primary and secondary outcomes, concluding that “no evidence is available to support treatment with ascorbic acid in adults with CMT1A”.

***Tagliavini Fabrizio - A randomized, double-blind pilot study versus placebo for the evaluation of the efficacy of doxycycline administered by oral route in patients affected by Creutzfeldt-Jakob disease***

Tagliavini and coll. were funded a project to assess the role of doxycycline in treating Creutzfeldt-Jacob disease, a fatal prion encephalopathy[4].Previous animal models and of preliminary observations in humans suggested that doxycycline might increase the survival time in comparison with historical series. The primary outcome of the study, in which 121 patients were randomized to doxycycline or placebo, was the survival time from randomization. The trial was stopped early for futility after an interim analysis showed the absence of superiority in survival time in the group receiving doxycycline compared to placebo (HR 1.1; 95% CI 0.8-1.7).

**Potentially beneficial and proof of concept**

***Ventura Alessandro - Randomized controlled double-blind vs. placebo multicentre study on the safety and effectiveness of thalidomide in the treatment of refractory Crohn’s disease and ulcerative colitis in children and adolescents***

The study coordinated by Ventura was aimed at assessing the effectiveness of thalidomide in inducing clinical remission in children with refractory Crohn disease [5]. In a multicenter, double-blind, placebo-controlled trial, 56 children with active Crohn disease despite immunosuppressive treatment, were randomized to thalidomide or placebo. At the end of the 8 week treatment, clinical remission was achieved by significantly more children in the thalidomide group (13/28 vs 3/26; risk ratio 4.0; 95%CI 1.2-12.5). Of the non-responders in the placebo group who were shifted to thalidomide, 11 of 21 (52.4%) reached remission at week 8. Moreover, mean duration of clinical remission in all children who received thalidomide was 181.1 weeks. The findings were strongly supportive of the efficacy of thalidomide in children with Crohn disease, even though the authors recognized that the sample size was inadequately powered to detect severe adverse events and that the study required replication before recommending the treatment in clinical practice.

***Olivieri Attilio – Imatinib Mesylate in the treatment of refractory extensive chronic Graft Versus Host Disease (cGVHD) with features Scleroderma-like***

The therapy of patients presenting steroid-refractory chronic graft-versus-host disease (SR-cGVHD) represents an unmet clinical need. The potential role of imatinib in this condition is still controversial; moreover, a major limitation in comparing study results is represented by the lack of standardized response criteria. In the project coordinated by Olivieri, the role of imatinib in SR-cGVHD was investigated in a prospective study that included 39 patients; the response was evaluated comparing different criteria[6]. After a median follow-up of 40 months, 28 patients were alive, with a 3-year overall survival and event-free survival of 72% and 46%, respectively. These findings, while suggesting imatinib as a valuable option for patients with SR-cGVHD, require to be confirmed in a larger randomized controlled trial.

References:

1. Visentini M, Ludovisi S, Petrarca A, et al. A phase II, single-arm multicenter study of low-dose rituximab for refractory mixed cryoglobulinemia secondary to hepatitis C virus infection. Autoimmun Rev 2011;10:714-9.
2. Fecarotta S, Romano A, Della Casa R, et al. [Long term follow-up to evaluate the efficacy of miglustat treatment in Italian patients with Niemann-Pick disease type C.](http://www.ncbi.nlm.nih.gov/pubmed/25888393) Orphanet J Rare Dis 2015;10:22.
3. Pareyson D, Reilly MM, Schenone A, et al. [Ascorbic acid in Charcot-Marie-Tooth disease type 1A (CMT-TRIAAL and CMT-TRAUK): a double-blind randomised trial.](http://www.ncbi.nlm.nih.gov/pubmed/21393063) Lancet Neurol 2011;10:320-8.
4. Haïk S, Marcon G, Mallet A, et al. [Doxycycline in Creutzfeldt-Jakob disease: a phase 2, randomised, double-blind, placebo-controlled trial.](http://www.ncbi.nlm.nih.gov/pubmed/24411709) Lancet Neurol 2014;13:150-8.
5. Lazzerini M, Martelossi S, Magazzù G, et al. [Effect of thalidomide on clinical remission in children and adolescents with refractory Crohn disease: a randomized clinical trial.](http://www.ncbi.nlm.nih.gov/pubmed/24281461) JAMA 2013;310:2164-73.
6. Olivieri A, Cimminiello M, Corradini P, et al. [Long-term outcome and prospective validation of NIH response criteria in 39 patients receiving imatinib for steroid-refractory chronic GVHD.](http://www.ncbi.nlm.nih.gov/pubmed/24152907)Blood 2013;122:4111-8.
