# Supplementary material for: Italian program for independent research on drugs: 10 year follow-up of funded studies in the area of rare diseases
Source: Orphanet J Rare Dis. 2016 Apr 12;11:36. doi: 10.1186/s13023-016-0420-4 (PMC4828875; doi:10.1186/s13023-016-0420-4)
Supplement: Additional file 7: Table S3. — Association of selected variables and clinical relevance of the published studies (n = 39). (DOCX 21 kb) [file 13023_2016_420_MOESM7_ESM.docx]

**Additional file 7: Table S3. Association of selected variables and clinical relevance of the published studies (n=39).**

|  | **PB + CN +CP** | | **PoC** | | **TOTAL** | **OR** | **95% CI** |
| --- | --- | --- | --- | --- | --- | --- | --- |
|  | N | % | N | % |  |  |  |
| **IF (categories)** | **16** | **100** | **23** | **100** | **39** |  |  |
| ≤5 | 5 | 31 | 11 | 48 | 16 | 1 |  |
| >5 | 11 | 69 | 12 | 52 | 23 | 2.0 | 0.5 - 7.7 |
| **Sample size (tertiles)** |  |  |  |  |  |  |  |
| 0-46 | 6 | 38 | 7 | 30 | 13 | 1 |  |
| 46-100 | 6 | 38 | 8 | 35 | 14 | 0.9 | 0.2 – 4.0 |
| 101+ | 4 | 25 | 8 | 35 | 12 | 0.6 | 0.1 - 2.9 |
| **Endpoint** |  |  |  |  |  |  |  |
| Surrogate endpoint | 8 | 50 | 17 | 74 | 25 | 1 |  |
| Clinical endpoint | 8 | 50 | 6 | 26 | 14 | 2.8 | 0.7 - 10.9 |
| **RCT vs. CT** |  |  |  |  |  |  |  |
| CT | 5 | 31 | 13 | 57 | 18 | 1 |  |
| RCT | 11 | 69 | 10 | 43 | 21 | 2.9 | 0.7 - 10.9 |
| **Treatment in control group** |  |  |  |  |  |  |  |
| **Total** | **11** | **100** | **10** | **100** | **21** |  |  |
| Active Control | 6 | 55 | 5 | 50 | 11 | 1 |  |
| Placebo/No treatment | 5 | 45 | 5 | 50 | 10 | 0.8 | 1.1 - 4.6 |

PB: potentially breakthrough findings; CN: conclusive negative findings; CP; conclusive positive findings; PoC: proof of concept studies; OR: Odds ratio; CI: Confidence intervals; IF: impact factor; RCT; randomized clinical trial; CT: clinical trial
